# Evaluating three internal fixation techniques for Pauwels III femoral neck fractures via finite element analysis

**DOI:** 10.1038/s41598-024-66638-1

**Published:** 2024-07-05

**Authors:** Ning Li, Kai-Yuan Cheng, Jixing Fan, Yu Li, Minghui Yang, Shiwen Zhu, Xieyuan Jiang

**Affiliations:** 1 Department of Orthopaedics and Traumatology, Beijing Jishuitan Hospital, Capital Medical University, No. 31 Xinjiekou East Street, Xicheng District, Beijing, 100035 China; 2 Department of Orthopedics, Peking University Third Hospital, Beijing, 100191 China; 3 College of Engineering, China Agricultural University, Beijing, 100083 China

**Keywords:** Femoral neck fracture, Cannulated compression screws (3CS), Biplane double-supported screw fixation (BDSF), Femoral neck system (FNS), Finite element analysis, Fracture repair, Trauma, Experimental models of disease

## Abstract

The selection of implants for fixing unstable femoral neck fractures (FNF) remains contentious. This study employs finite element analysis to examine the biomechanics of treating Pauwels type III femoral neck fractures using cannulated compression screws (3CS), biplane double-supported screw fixation (BDSF), and the femoral neck system (FNS). A three-dimensional model of the proximal femur was developed using computed tomography scans. Fracture models of the femoral neck were created with 3CS, BDSF, and FNS fixations. Von Mises stress on the proximal femur, fracture ends, internal fixators, and model displacements were assessed and compared across the three fixation methods (3CS, BDSF, and FNS) during the heel strike of normal walking. The maximum Von Mises stress in the proximal fragment was significantly higher with 3CS fixation compared to BDSF and FNS fixations (120.45 MPa vs. 82.44 MPa and 84.54 MPa, respectively). Regarding Von Mises stress distribution at the fracture ends, the highest stress in the 3CS group was 57.32 MPa, while BDSF and FNS groups showed 51.39 MPa and 49.23 MPa, respectively. Concerning implant stress, the FNS model exhibited greater Von Mises stress compared to the 3CS and BDSF models (236.67 MPa vs. 134.86 MPa and 140.69 MPa, respectively). Moreover, BDSF displayed slightly lower total displacement than 3CS fixation (7.19 mm vs. 7.66 mm), but slightly higher displacement than FNS (7.19 mm vs. 7.03 mm). This study concludes that BDSF outperforms 3CS fixation in terms of biomechanical efficacy and demonstrates similar performance to the FNS approach. As a result, BDSF stands as a dependable alternative for treating Pauwels type III femoral neck fractures.

With an aging population and extended life expectancy, the occurrence of hip fractures among the elderly has risen rapidly in recent decades. Globally, approximately 1.5 million hip fractures are recorded yearly, a number projected to reach 6.3 million by 2050^1^. Among these, femoral neck fractures (FNF) constitute half of all hip fractures and pose a substantial public health challenge, entailing significant socioeconomic costs^2^. Pauwels Type III femoral neck fractures, due to pronounced vertical shear forces, present difficulties in achieving sufficient stability, leading to complications such as end displacement, bone nonunion, and femoral head necrosis^3^. Notably, reports indicate a nonunion incidence of 16–59% for Pauwels type III femoral neck fractures^4^.

The selection of implants for fixing femoral neck fractures remains a contentious issue in addressing unstable cases. Presently, common internal fixation methods involve cannulated screw fixation^5^. The standard approach involves a construct of three inverted triangle cannulated compression screws (3CS), which offers less invasiveness and better preservation of blood supply. However, biomechanical stability is relatively lower, potentially resulting in issues like femoral neck shortening and varus collapse^6^. The calcar femorale, a supportive structure for the femoral neck, plays a crucial role in load distribution and mechanical support within the proximal femur. Thus, restoring this structure's mechanics is paramount in femoral neck fracture treatment. Some study introduced biplane double-supported screw fixation (BDSF) to restore the calcar femorale. In the BDSF model, the inferior and middle screws are angled differently in the coronal plane to buttress the calcar^7^.

The femoral neck system (FNS), a novel minimally invasive implant designed for dynamically fixing femoral neck fractures, combines angular stability with minimally invasive surgery benefits^8^. Nevertheless, the FNS has drawbacks compared to cannulated screw fixation. It necessitates a 5–7 cm lateral incision near the greater trochanter, potentially leading to increased soft tissue exposure^9^. Furthermore, cost-effectiveness greatly impacts patient adherence, especially in developing nations. The FNS incurs significantly higher costs than cannulated screws. In our perspective, BDSF harmonizes the benefits of minimally invasive procedures and enhanced biomechanical stability. Whether BDSF offers biomechanical advantages over FNS and 3CS fixation for Pauwels type III femoral neck fractures remains uncertain. This study's goal is to assess the biomechanical efficacy of 3CS, BDSF, and FNS in treating Pauwels type III femoral neck fractures. This endeavour aims to establish a theoretical foundation and reference for clinically managing femoral neck fractures.

## Materials and methods

### Finite element model establishment

The femur's geometric model was derived from a three-dimensional representation of a left fourth-generation composite femur (MODEL3405#, Pacific Research Laboratories Vashon, WA). A 64-slice spiral CT scan (GE, USA) was used to capture the proximal femur's details, and the resulting data was saved in the Digital Imaging and Communications in Medicine (DICOM) format. The femur data was then imported into Mimics 17.0 software (Materialise, Belgium) to reconstruct a three-dimensional model of the proximal femur based on the CT images. Surface irregularities (spikes, intersections, etc.) on the 3D proximal femur model were corrected using Geomagic Studio 12.0 software (Raindrop Inc., USA). After addressing the model's surface irregularities, a smooth 3D solid model was created and brought into the SolidWorks program (Dassault Systemes SolidWorks Corp., USA). To simulate Pauwels type III femoral neck fractures, consistent with prior literature^10,11^, we designed fractures with 70° angles using SolidWorks 2017 software.

**Figure 1:**
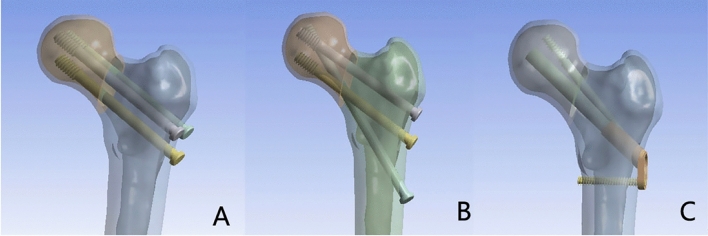
Illustration of the three femoral fracture models with different internal fixation methods. (**A**) Femoral neck fracture model with 3CS fixation; (**B**) Femoral neck fracture model with BDSF fixation; (**C**) Femoral neck fracture model with FNS fixation.

Following DePuy Synthes' cannulated screw specifications (West Chester, PA, USA), screws with a threaded portion diameter of 7.3 mm and a length of 16 mm were employed, alongside a non-threaded section with a diameter of 4.8 mm. For 3CS model, three screws were placed in a parallel symmetry, forming an isosceles inverted triangle^12^; for BDSF model, the distal and the middle screws are calcar-buttressed with coronal inclinations of 150°–165° and 130°–140°, respectively^13^; for the FNS model, a 10 mm diameter sliding hip screw was positioned at a 130° angle to the locking plate. At the proximal FNS end, a 6.4 mm diameter locking anti-rotational screw was placed at a 7.5° angle to the sliding hip screw, and a 5-mm hole was created at the distal end^14^. The 3CS, BDSF, and FNS constructs were virtually incorporated into the proximal femur (Fig. 1). Subsequently, the models underwent analysis in ANSYS Workbench 14.5 (ANSYS Inc., Canonsburg, PA).

Solid models were discretized into four-node tetrahedral elements within ANSYS Workbench. We achieved convergence in our finite element analysis by systematically refining the mesh. Starting with a coarse mesh, we gradually reduced the element size while observing variations in critical output measures like stress and displacement. Convergence was established when successive mesh refinements yielded changes in these measures below a predetermined percentage. In line with standard practice, we deemed the model converged when the variation in the quantity of interest was below 1% between mesh refinements. To ensure model accuracy, convergence tests were conducted to ascertain the optimal maximum element size. Post-convergence assessment, the mesh size was determined to be 2 mm^15^.

In this study, all materials were treated as homogeneous, isotropic, and linearly elastic^16^. Material properties for the femur and implant components used in the models were outlined in Table 1^17,18^. Contact between the internal fixation screw and femur was established using established and approved contact setup techniques from prior research^19,20^. Friction contact was applied on the fracture surface, utilizing a friction coefficient of 0.46^21,22^.

**Table 1:** Material properties used in the simulations in this study.

Material	Young’s modulus (Mpa)	Poisson’s ratio
Cortical bone	17,000	0.33
Cancellous bone	1000	0.3
DHS (Ti–6Al–7NB)	110,000	0.35

### Boundary and loading conditions

For the boundary conditions, the femur's distal end was constrained in all degrees of freedom. The applied loading simulated the forces experienced during normal walking's heel strike phase^23^. Specifically, a joint reaction force of 2967.7 N was exerted on the femoral head (equivalent to 4.2 times body weight). The joint reaction force was applied to the femoral head at an angle of 16° medially and 11° anteriorly relative to the femur's vertical axis, as reported in the previous literature^24^. To mitigate bending moments at the proximal femur, an abductor force was applied at the greater trochanter, oriented at an angle of 25° superiorly and 30° posteriorly relative to the femoral shaft's axis^25,26^. This abductor muscle load, amounting to 1288.3 N, was directed at the greater trochanter (equivalent to 1.9 times body weight).

### Evaluation criteria

In the finite element analysis, Von Mises stress was assessed on the proximal femur, fracture ends, internal fixators, and model displacements. These parameters were compared for the three fixation methods (3CS, BDSF, and FNS) under conditions simulating the heel strike of normal walking.

## Results

### Von Mises stress in the proximal femur

**Figure 2:**
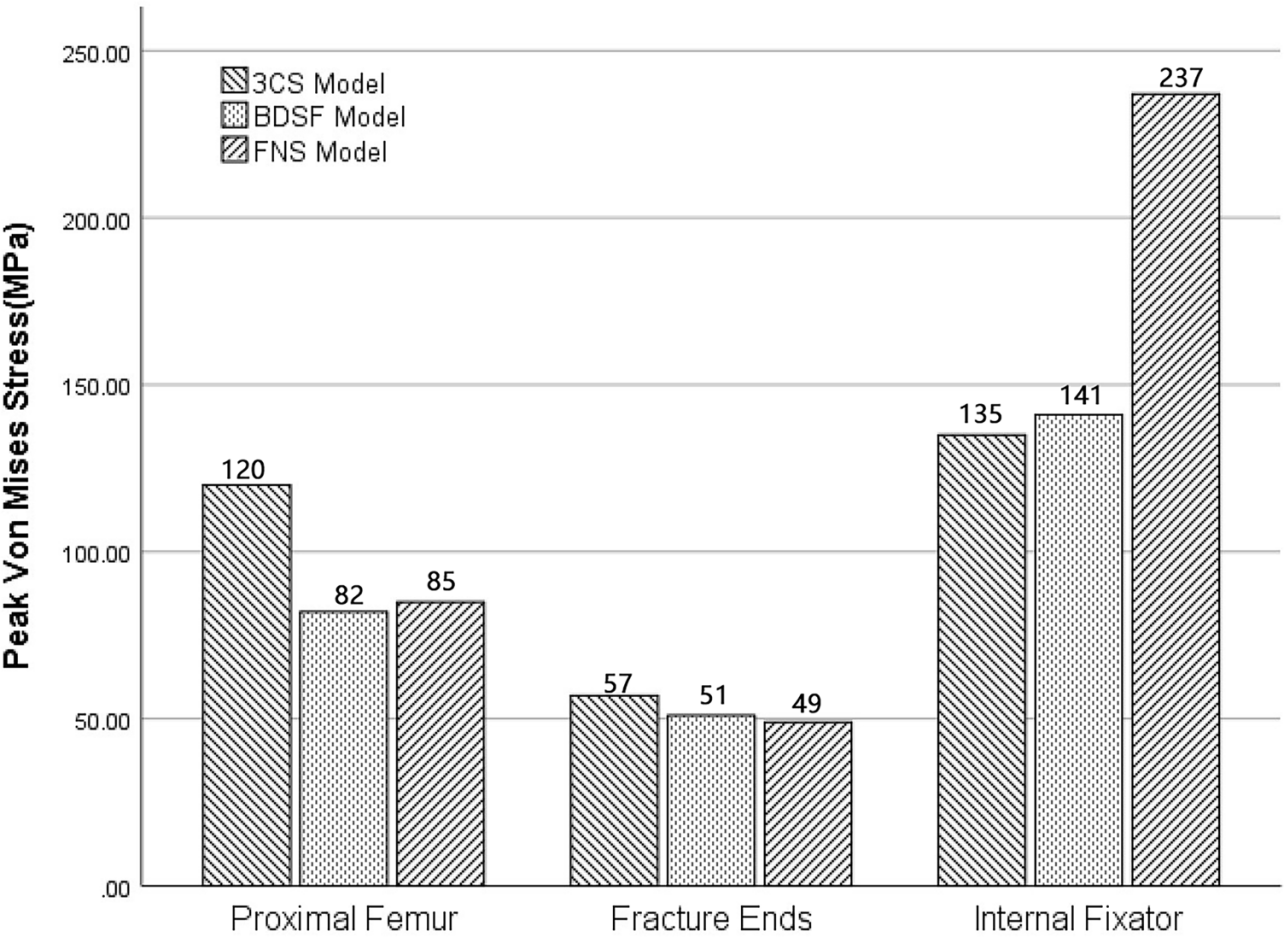
Comparison of peak Von Mises stress on the proximal femur, fracture ends, and internal fixators among the three fixation styles (3CS, BDSF, and FNS) during heel strike in normal walking.

**Figure 3:**
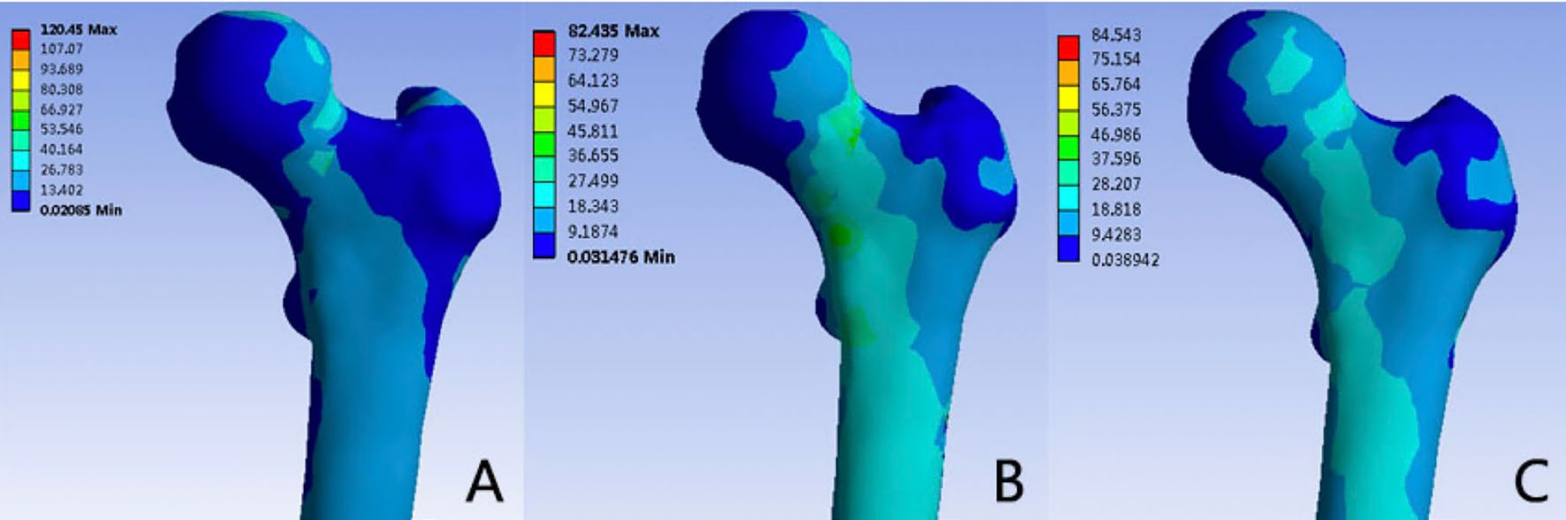
Distribution of Von Mises stress (MPa) on the proximal femur: (**A**) 3CS model; (**B**) BDSF model; (**C**) FNS model.

The distribution of von Mises stress in the proximal femur is illustrated in Figs. 2 and 3. Across the three models, stress concentration was evident at the fracture point and in proximity to the lesser trochanter. The highest von Mises stress on the femur was recorded in the 3CS group at 120.45 MPa, followed by 82.44 MPa in the BDSF group and 84.54 MPa in the FNS group. Notably, the 3CS fixation exhibited notably higher peak stress compared to the other two methods.

### Von Mises stress of implant

**Figure 4:**
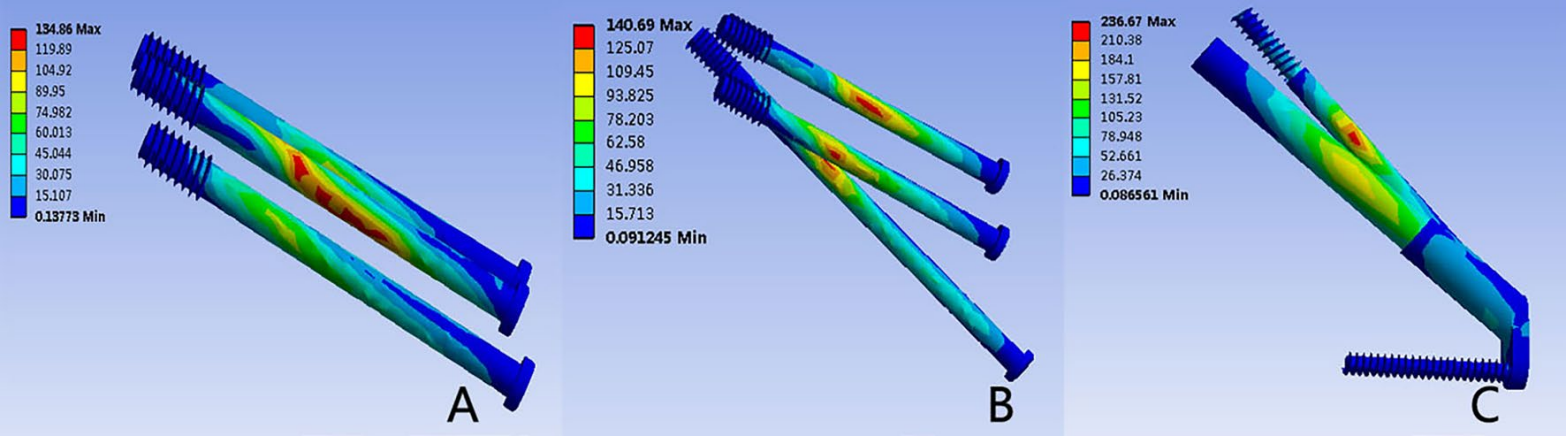
Distribution of Von Mises stress (MPa) on the implant: (**A**) 3CS model; (**B**) BDSF model; (**C**) FNS model.

**Figure 5:**
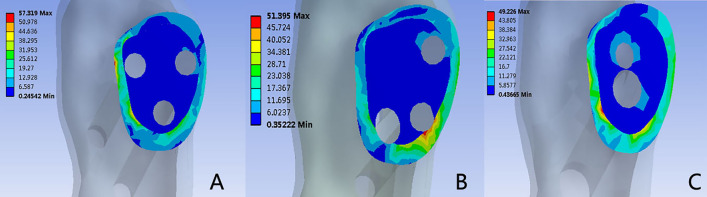
Distribution of Von Mises stress (MPa) on the fracture ends: (**A**) 3CS model; (**B**) BDSF model; (**C**) FNS model.

Figures 4 and 5 showcase the distribution of von Mises stress within the three internal fixation models. For each model, stress concentration was observed along the middle surface of the screw near the fracture line, displaying even distribution along the screw's length. The 3CS and BDSF models demonstrated similar peak von Mises stress, measuring 134.86 MPa and 140.69 MPa, respectively. In contrast, the FNS model exhibited the highest von Mises stress among the three, reaching 236.67 MPa.

### Von Mises in the fracture ends

Figures 2 and 5 visualize the von Mises stress distribution at the fracture ends. In all models, stress concentration occurred at the intersection of internal fixation and the fracture surface. The region of elevated stress was notably higher below the femoral neck than above it. The maximal von Mises stress in the 3CS group registered at 57.32 MPa, while the BDSF and FNS groups recorded 51.39 MPa and 49.23 MPa, respectively. The 3CS fixation displayed greater peak stress compared to the other two surgical techniques.

### Model displacement

**Figure 6:**
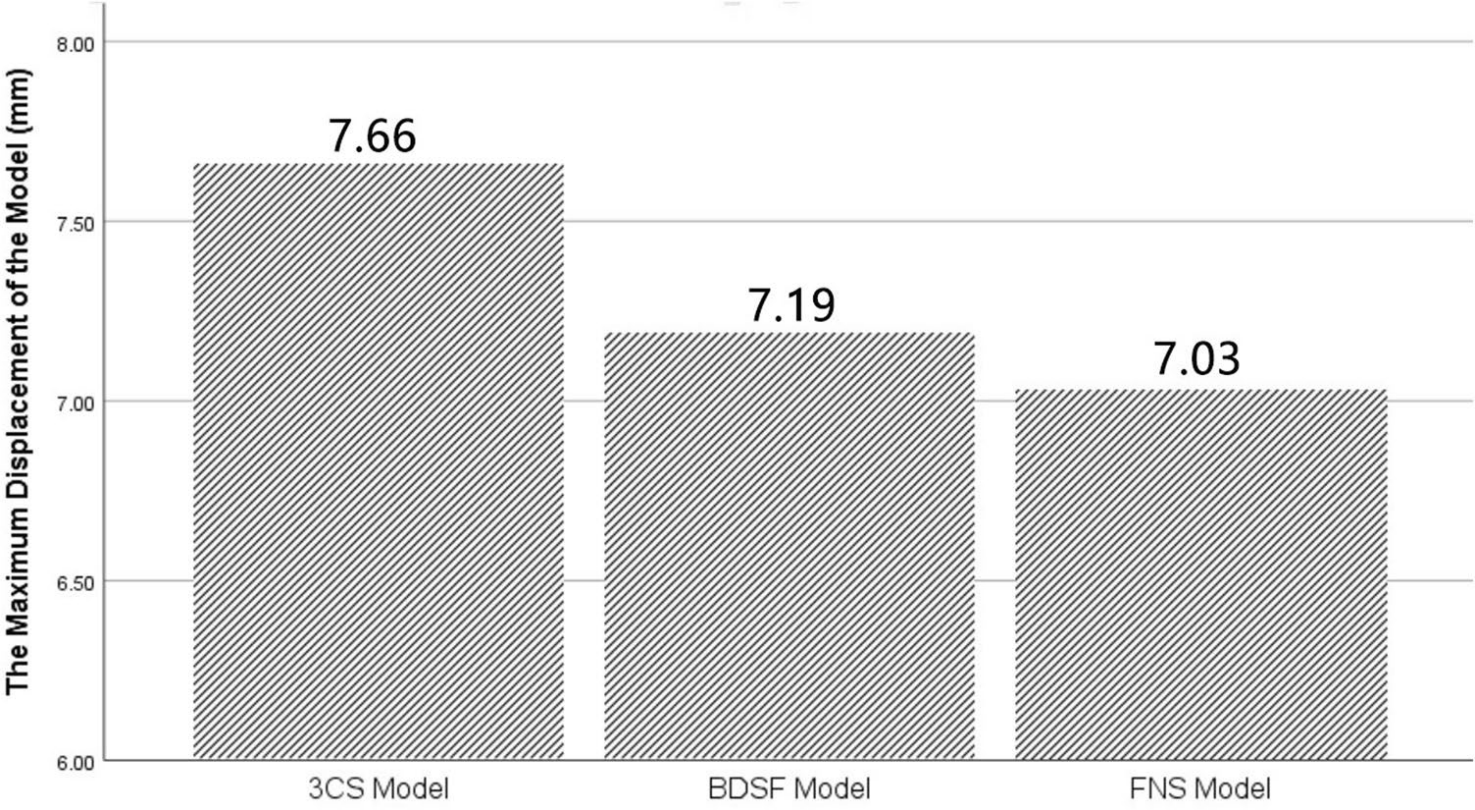
Evaluation and comparison of model displacements among the three fixation styles (3CS, BDSF, and FNS) during heel strike in normal walking.

**Figure 7:**
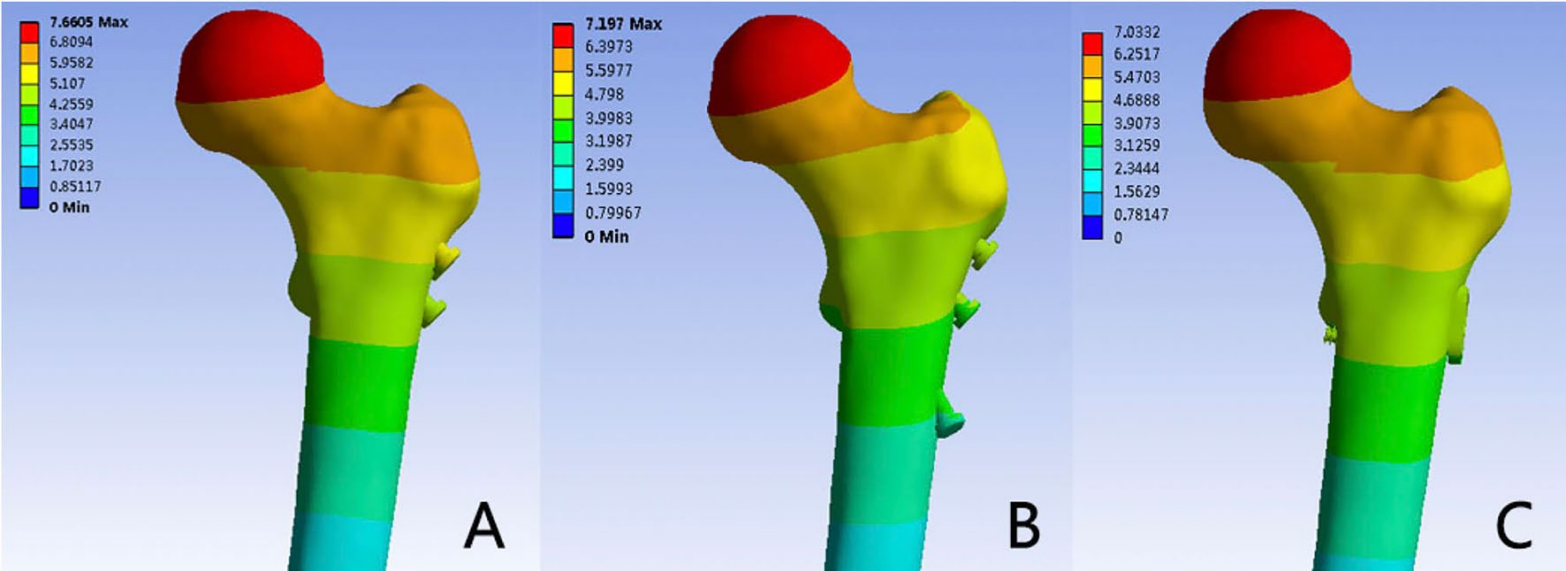
Distribution of displacements (mm) in the three models: (**A**) 3CS model; (**B**) BDSF model; (**C**) FNS model.

Figures 6 and 7 provide insight into model displacement across the three models. Maximum displacements were localized at the upper portion of the femoral head in all instances. Among the models, the 3CS configuration exhibited the greatest maximum displacement, measuring 7.66 mm, followed by the BDSF model at 7.19 mm, and the FNS model at 7.03 mm.

## Discussion

Currently, the most effective surgical approach for femoral neck fractures remains a matter of debate. The 3CS technique has gained widespread use for treating these fractures. However, the 3CS method struggles to maintain a secure grip on fracture ends and lacks perpendicular sliding compression along the fracture line. A meta-analysis indicated that when FNFs are managed with SHS or CS, the complication rate can be high, with reported reoperation rates of 18–27%^27^ and a failure rate of up to 43%^28^. On the other hand, the FNS represents a novel minimally invasive implant designed to enhance angular stability. Numerous studies suggest that FNS exhibits superior biomechanical properties compared to 3CS screw fixation for addressing Pauwels type III fractures^9,29^. Nevertheless, FNS involves a lateral incision of 5 to 7 cm near the greater trochanter, potentially increasing soft tissue exposure. In the meantime, a retrospective study of 62 FNF patients treated with FNS fixation reported that 10 patients (16.1%) developed early failure of FNS. The study concludes that age, Garden classification, and other examined factors do not significantly influence the risk of early failure of FNS in treating FNF^30^. Furthermore, cost-effectiveness weighs heavily on patient compliance, particularly in developing countries, where FNS proves significantly pricier than conventional cannulated screw (CC screw) fixation. Hence, alternative CS fixation configurations could prove valuable for femoral neck fractures in such contexts. The introduction of BDSF, designed to substantially enhance cannulated screw osteosynthesis stability via innovative biomechanics, emerges as a solution^7^. BDSF provides stability for FNF, demonstrating a high rate of bone union with low incidence of nonunion and avascular necrosis over a 5-year period^31^. The objective of our study was to assess the biomechanical performance of 3CS, BDSF, and FNS for Pauwels type III femoral neck fractures.

Our approach employed computer simulations through the finite element method (FEM) to explore the biomechanical performance of various surgical methods for Pauwels III femoral neck fractures. Analyzing displacement of the proximal femur and the internal fixation device after force application offered insight into internal fixation model stability. Findings revealed that the 3CS model exhibited the greatest maximum displacement and highest peak von Mises stress in the proximal femur and fracture end, outstripping the BDSF and FNS models. The location of the maximum von Mises stress in the femur is typically associated with the areas of highest load concentration and weakest structural integrity, and located at the fracture point and in proximity to the lesser trochanter in our study. The elevated stress concentration of 3CS at the fracture point and proximal femur suggests that 3CS parallel configuration may induce greater mechanical loads on the bone, potentially affecting the healing process and risk of implant failure^20^. A recent study by Liu et al. emphasized the importance of stress distribution in predicting the risk of secondary fractures and implant-related complications. The higher stress in the 3CS group could potentially increase the risk of excessive micro-movements at the fracture site, hindering callus formation and leading to delayed union and nonunion^32^. This study also confirmed that FNS and BDSF really decreased the stress at the fracture point. We are the first research to report a comparative study through finite element analysis of three types of 3CS, BDSF and FNS simultaneously. These results suggest BDSF's superiority over the traditional 3CS technique and its comparable biomechanical performance to FNS. This aligns with previous research indicating that BDSF offers enhanced stability and superior fixation strength, both pivotal for successful outcomes in femoral neck fracture treatment^33^. In contrast, the 3CS technique, though widely utilized, has demonstrated higher stress concentrations at the fracture site, potentially leading to increased complications like non-union or avascular necrosis (AVN). Our discovery of elevated von Mises stress within the 3CS cohort corroborates existing literature on this matter^34^. Clinical indicators such as fracture union rates and occurrences of AVN further support our observations. Literature highlights BDSF's favorable outcomes, with high rates of fracture union and minimal occurrences of AVN, in comparison to those associated with 3CS^31,33^. These clinical benchmarks play a crucial role in confirming the biomechanical efficacy of internal fixation methods.

FNF often coincide with calcar femorale disruption, a pivotal weight-bearing structure in the proximal femur responsible for transmitting stress from the femoral head and neck to the femoral shaft^35^. Femoral neck fractures often coincide with calcar femorale disruption, a pivotal weight-bearing structure in the proximal femur responsible for transmitting stress from the femoral head and neck to the femoral shaft^36^. The 3CS model approached the bone's yield strength, suggesting heightened vulnerability compared to BDSF and FNS, where stress levels were considerably below the yield strength. Consequently, 3CS fixation poses a higher risk of proximal femur failure than BDSF and FNS alternatives. Generally, diminishing the implant's peak von Mises stress reduces the potential for implant failure during daily loading. In our analysis, stresses primarily concentrated at the screw's mid-surface near the fracture line, evenly distributed along the screw in each model. Both the 3CS and BDSF models exhibited similar peak von Mises stress^31^, measuring 120.34 MPa and 114.04 MPa, respectively. The mechanical performance of internal fixation devices is crucial for ensuring stability and promoting fracture healing. Certain geometrical designs, including non-parallel configurations and locking systems, are proposed to reduce the stress concentration at the fracture site^37^. Under identical loading conditions, the FNS model experienced the highest von Mises stress compared to the 3CS and BDSF models (236.67 MPa, 134.86 MPa, and 140.69 MPa, respectively). While the peak stress observed in the FNS model was below the ultimate strength (approximately 921 MPa for Ti–6Al–7NB alloy), repeated loading might lead to screw fatigue failure^38^.

In the meantime, patient-specific factors such as age, comorbidities, bone quality and activity level also plays an essential in the choice of fixation method. Younger patients may have better bone quality and healing potential, which can influence the choice of a more conservative fixation method. In contrast, older patients may benefit from methods that provide immediate stability due to poorer bone quality and healing capacity^39^. Patients with comorbid conditions such as diabetes or osteoporosis may have impaired bone healing, necessitating a fixation method that provides enhanced stability and promotes bone growth^40^. The density and quality of the bone can significantly impact the choice of fixation. Poor bone quality may require augmentation techniques or fixation methods that distribute load more effectively^41^. The expected post-operative activity level is crucial. Active patients may require a more robust fixation to withstand early mobilization and weight-bearing in FNF^42^.

This study possesses certain limitations. Firstly, both the femur and implants are anisotropic materials, yet we simplified them as homogenous, isotropic, and elastic for analysis simplicity. In FEA, simplifications have the potential to either underestimate or overestimate stress distributions and mechanical responses due to the inability to accurately capture the true stress–strain relationship under various loading conditions. Secondly, the influence of soft tissues, including muscles and skin around the femur, on post-internal fixation forces wasn't considered. Moreover, neglecting soft tissues may result in an incomplete representation of the physiological environment, thereby affecting the accuracy of predicted stress and displacement fields. Additionally, our study relied on FEM with simulated reconstructed models based on CT images, while actual surgical procedures are more intricate. This preliminary investigation underscores the need for larger-scale clinical research to provide more comprehensive comparisons. The implications on generalizability are noteworthy, as the model may not fully encompass the complex interactions present in real-world settings, potentially leading to disparate outcomes in clinical practice. To enhance the validity and generalizability of FEA studies, it is imperative to incorporate more advanced material models that consider the nonlinear, anisotropic, and time-dependent properties of bone, as well as to integrate the effects of soft tissues. Furthermore, future studies could benefit from comparing FEA results with experimental or clinical data to validate the model and enhance its applicability across a broader spectrum of conditions.

In summary, our study provides conclusive evidence for clinical decision-making: BDSF outperforms 3CS fixation in terms of biomechanical efficacy and demonstrates similar performance to the FNS approach. An appropriate choice of fixation methods could contribute to fracture healing and decrease postoperative complications and implant failure rates. Moreover, the choice of fixation methods also depends on various factors including patient-specific factors. The translation of biomechanical performance translates into clinical outcomes and patient requires further study.

## Conclusion

This study conducted a biomechanical assessment of 3CS, BDSF, and FNS fixation methods in addressing Pauwels type III femoral neck fractures. The outcomes indicate that BDSF demonstrates enhanced biomechanical effectiveness compared to 3CS fixation and comparable performance to FNS methods. Consequently, BDSF emerges as a dependable alternative for treating Pauwels type III femoral neck fractures, particularly in developing countries. These insights offer a theoretical foundation for guiding clinical approaches to femoral neck fractures.

## Data Availability

All data generated and analysed during this study are included in this article.

## Abbreviations

3CS: Cannulated compression screws
BDSF: Biplane double-supported screw fixation
FNS: Femoral neck system
DICOM: Digital Imaging and Communications in Medicine
FEM: Finite element method
FNF: Femoral neck fracture
